# Estimating the Influenza Vaccine Effectiveness against Medically Attended Influenza in Clinical Settings: A Hospital-Based Case-Control Study with a Rapid Diagnostic Test in Japan

**DOI:** 10.1371/journal.pone.0052103

**Published:** 2013-01-11

**Authors:** Motoi Suzuki, Hiroyuki Yoshimine, Yoshitaka Harada, Naho Tsuchiya, Ikumi Shimada, Koya Ariyoshi, Kenichiro Inoue

**Affiliations:** 1 Department of Clinical Medicine, Institute of Tropical Medicine, Nagasaki University, Nagasaki, Japan; 2 Inoue Hospital, Shunkaikai, Nagasaki, Japan; Menzies School of Health Research, Australia

## Abstract

**Background:**

Influenza vaccine effectiveness (VE) studies are usually conducted by specialized agencies and require time and resources. The objective of this study was to estimate the influenza VE against medically attended influenza using a test-negative case-control design with rapid influenza diagnostic tests (RIDT) in a clinical setting.

**Methods:**

A prospective study was conducted at a community hospital in Nagasaki, western Japan during the 2010/11 influenza season. All outpatients aged 15 years and older with influenza-like illnesses (ILI) who had undergone RIDT were enrolled. A test-negative case-control design was applied to estimate the VEs: the cases were ILI patients with positive RIDT results and the controls were ILI patients with negative RIDT results. Information on patient characteristics, including vaccination histories, was collected using questionnaires and medical records.

**Results:**

Between December 2010 and April 2011, 526 ILI patients were tested with RIDT, and 476 were eligible for the analysis. The overall VE estimate against medically attended influenza was 47.6%, after adjusting for the patients' age groups, presence of chronic conditions, month of visit, and smoking and alcohol use. The seasonal influenza vaccine reduced the risk of medically attended influenza by 60.9% for patients less than 50 years of age, but a significant reduction was not observed for patients 50 years of age and older. A sensitivity analysis provided similar figures.

**Conclusion:**

The test-negative case-control study using RIDT provided moderate influenza VE consistent with other reports. Utilizing the commonly used RIDT to estimate VE provides rapid assessment of VE; however, it may require validation with more specific endpoint.

## Introduction

Vaccination plays a central role in the influenza control program [1], [2]. However, the effectiveness of the influenza vaccine substantially varies from season to season because of the antigenic drift of the circulating strain [1], [3]. In the USA, the vaccine effectiveness (VE) against medically attended influenza ranges from 10% (2004/05 season) to 52% (2006/07 season) in residents who are recommended for vaccination [4]. The influenza VE also varies from country to country. Recent studies have shown that the estimate of VE against influenza A (H1N1) 2009 was 49% in Australia [5] and 93% in Canada [6]. Regional variations of VE may be explained by the differences in the vaccine products (e.g., adjuvanted *vs.* nonadjuvanted, monovalent *vs.* trivalent, or activated *vs.* inactivated) and pre-existing anti-influenza immunity among the population [7]. Monitoring the region-specific and season-specific VE is therefore essential for an efficient influenza control program.

In Japan, the influenza season generally occurs between December and April [8]. Before influenza activity begins, the Ministry of Health, Labor and Welfare (MHLW), the National Institute of Infectious Diseases (NIID) and the district health authorities conduct population-based serological surveys and release the data on serum-hemagglutinin-inhibition antibody titers (HI titer) against vaccine strains for promoting vaccination [9]. Furthermore, the seroconversion rate after vaccination is provided by domestic vaccine manufacturers. However, a high HI titer does not necessarily translate into actual protection against the circulating strain [1], [10], [11], and only a few studies have estimated the clinical effectiveness of vaccination on the risk of disease among Japanese population [12]–[14]. No official influenza VE monitoring system has been established in Japan.

As a randomized controlled trial of a licensed vaccine is unethical, influenza VEs are estimated using observational studies in many settings [1]. The test-negative case-control study has recently been recognized as an efficient method of estimating the VE [15]. In this design, samples are collected from patients with influenza-like illnesses (ILI), and the VE is estimated comparing the vaccination status of influenza positives, commonly assessed by RT-PCR, with that of influenza negatives. Although this design gives reliable VE estimates, only limited laboratories can perform RT-PCR testing of hundreds of clinical samples. Thus, clinicians and patients have been obliged to wait for reports from expert agencies.

In order to rapidly assess the VE against medically attended influenza, we evaluated the use of rapid influenza diagnostic tests (RIDT) results as an alternative to RT-PCR. The use of low-sensitivity tests tends to underestimate the true VEs [16], [17]; however, RIDT has advantages for its rapid reporting and large dataset since they are widely used in clinics and hospitals, especially in resource-rich settings such as Japan. To test the feasibility of the test-negative case-control design with RIDT as a simplified VE study in a real clinical setting, we conducted a study at a medium-size busy community-hospital in Nagasaki, western Japan during the 2010/11 influenza season.

## Methods

### Ethics

The study was approved by the Institutional Review Board (IRB) at Inoue Hospital, Nagasaki, and the IRB of the Institute of Tropical Medicine at Nagasaki University. Verbal informed consent was obtained from all participants or their guardians; the requirement for obtaining written consent was waived by both IRBs due to its observational nature without any deviation from the current medical practice. Our hospital doctors informed the study objectives and methods to eligible patients and their guardians verbally during their consultations. We also provided the necessary information to patients and their guardians using a standardized questionnaire sheet and a poster presentation at the outpatient department. Anonymized data were used for the analysis.

### Study setting and enrollment criteria

A prospective, hospital-based case-control study was conducted in Nagasaki. Inoue Hospital is a community-based private hospital located in the center of the city. The hospital has 112 beds that provide primary and secondary care for mainly adolescents and adults; the number of pediatric patients is small because the hospital does not have a pediatric department. Approximately 40% of outpatients are referred cases, while the majority of first visit patients with mild symptoms (e.g., cough and fever) arrive without referral letters. Because of the universal health coverage in Japan, 70% of the medical costs for people who are less than 70 years of age and 80–90% of the medical costs for people who are 70 years of age and older are covered by insurance in the private and public sectors [18]. We thus expect that the characteristics of the patients who visit this hospital with ILI symptoms are not distinct from those patients who visit neighboring clinics.

The study period was from December 20, 2010, through April 30, 2011. A standardized questionnaire was distributed to all new outpatients regardless of their ILI conditions during the study period. Patients and their care givers were asked to fill in the form before the consultation. All patients (not necessarily new patients) aged 15 years and older who visited the outpatient department (OPD), presented with ILI, and had been administered the RIDT were enrolled in the study. Pediatric patients were not included because the number of patients was limited. A case was defined as ILI if the patient showed the following: 1) at least one sign of cough, runny nose, sore throat, headache, myalgia or fatigue; 2) a sudden fever; and 3) a body temperature of ≥37.1°C at the first visit. A case was excluded if the testing was performed more than five days after the disease onset. In patients who had multiple episodes, only the first or influenza positive episode was included in the analysis.

A commercial RIDT kit (RapidTesta Flu II, Sekisui Medical, Japan) was used to identify influenza A- and B-positive cases throughout the study period. According to the manufacturer's instructions, the sensitivity and specificity of the kit compared with viral culture were 93.9% and 98.9% for influenza A (H1N1) 2009, 94.3% and 100% for all influenza A strains, and 85.2% and 100% for influenza B; however, such high sensitivities cannot be expected in community settings [19]. The RIDT was ordered by clinicians based on their judgment and performed by skilled nurses or laboratory technicians.

### 2010/11 season influenza vaccines in Japan

During the vaccination campaign between October 1, 2010, and March 31, 2011, all children less than 13 years of age were recommended to receive 2 doses of the 2010/11 season vaccine, and others were recommended to receive one dose by the MHLW. The standardized maximum cost of the vaccine was 3,600 yen (about 45 USD); however, the people in high-risk groups, including the elderly, were partially or fully subsidized by the local government [20].

In Japan, two types of influenza vaccines were available in the season: the trivalent inactivated 2010/11 seasonal influenza vaccine (TIV), which included the influenza A (H1N1) 2009 strain and the monovalent inactivated influenza A (H1N1) 2009 vaccine (MIV). The TIVs and MIVs were produced by four domestic manufacturers (Denka Seiken, Tokyo; Kaketsuken, Kumamoto; Kitasato Institute, Tokyo; and Biken, Suita). Another imported monovalent AS03 adjuvanted vaccine (Arepanrix, GlaxoSmithKline) was also available. However, before starting the vaccination campaign, the MHLW recommended using TIVs instead of MIVs, especially for elderly people [20]. In fact, Daiichi Sankyo Co., a wholesaler of vaccine products of the Kitasato Institute, had sold only TIVs to clinics and hospitals (personal communication), and based on our survey, MIVs had not been used in our hospital and neighboring clinics. Therefore, although the patients in our study were not asked which type of vaccine had been administered to them, we reasonably expected that people who reported being vaccinated during the 2010/11 season had been vaccinated with TIVs.

### Estimated vaccination coverage and sample size

According to the estimate by the National Epidemiological Surveillance of Vaccine-Preventable Diseases, the influenza vaccination coverage during the 2010/11 season was 45% among population aged > = 15 years: 45% and 44% among those aged <50 years and > = 50 years, respectively [21]. Assuming that the vaccination coverage among our source population was 50%, at a power of 80%, 111 (2 controls per case) to 148 (1 control per case) influenza positive cases were required to detect the vaccine effectiveness of 50%.

### Data collection and statistical analysis

Demographic data, clinical information and vaccination status were collected from questionnaires and electronic medical charts. The vaccination history was documented based on patient/family recall but not confirmed objectively because our hospital and neighboring clinics were not systematically recording the name of vaccinated people. The presence of a chronic condition was defined if a patient was taking any medications for more than three months.

We used the test-negative case-control study design for estimating VEs: the cases were all ILI episodes that were positive for influenza A and/or B by RIDT, and the controls were all ILI episodes that were negative for both influenza A and B. The characteristics of the study patients were compared by outcome categories. The patients' ages were categorized into four groups: 15–19 years, 20–49 years, 50–64 years and 65 years and above. The VEs against influenza were calculated as 1 – odds ratio (OR). Logistic regression models were used to estimate the unadjusted and adjusted ORs. All potential confounders were included in the models.

In our dataset, the number of missing values was not negligible in certain variables such as the vaccination history (∼15%), smoking (∼30%) and alcohol-drinking status (∼30%). Excluding the incomplete episodes from the dataset could have led to bias because the missing values were not completely at random [22]. We instead coded those missing values as “unknown status” and included all patients for our primary analysis (missing-indicator method) [23]. Then, a sensitivity analysis was performed; the VEs were estimated using episodes with complete data (complete-case analysis). All statistical tests were performed using STATA 11.2 (STATA Corp., USA).

## Results

**Figure 1:**
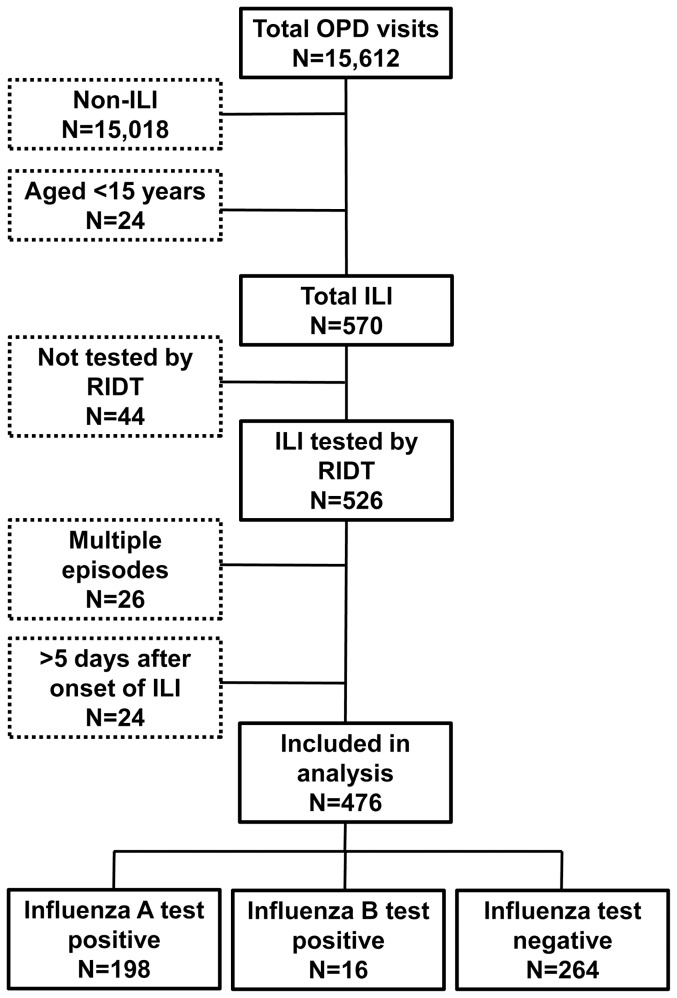
Inclusion and exclusion criteria for study participants in Nagasaki, Japan, December 2010–April 2011.

**Figure 2:**
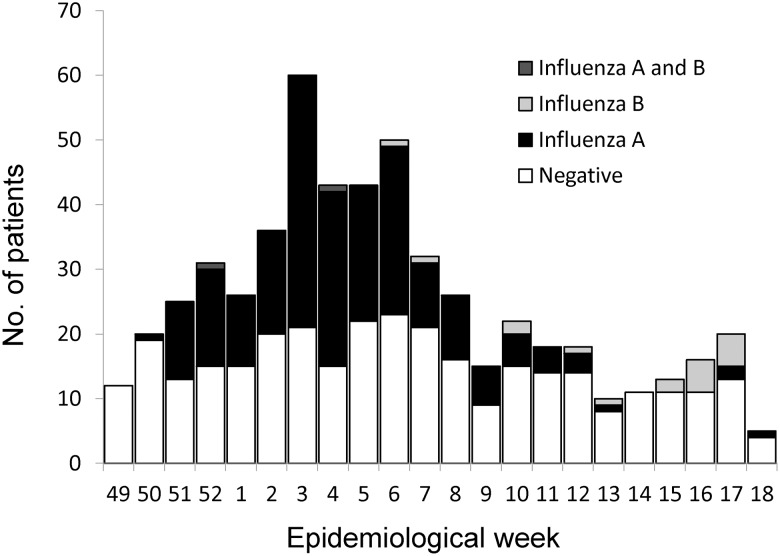
Numbers of influenza positives and negatives by week of hospital visit.

During the study period, 15,612 patients visited the outpatient department. Among them, 570 patients aged 15 years and over presented with ILI, and 526 (92.3%) were tested for influenza by RIDT. Compared with the patients who were tested by RIDT, patients not tested by RIDT (N = 44) were older (39.8 years *vs.* 47.6 years, p = 0.01), more had underlying conditions (33% *vs.* 48%, p = 0.049), and less vaccinated (31.8% *vs.* 11.4%, p<0.001). The hospitalization rate was similar between tested patients and untested patients (0.8% *vs.* 0%, p = 1.0). After application of the exclusion criteria, 476 patients were included in the analysis (Figure 1). Among all 476 that were tested, 196 (41.2%) were positive only for influenza A, 14 (2.9%) were positive only for influenza B, 2 (0.4%) were positive for both influenza A and B, and the rest were negative for both tests. The number of influenza A-positive patients reached the peak at week 3 in 2011, and influenza B-positive patients were seen after week 6 (Figure 2).

The characteristics of the study patients by outcome status are shown in Table 1. The majority of our patients were 20–49 years of age. The age distribution was similar between cases and controls. Most of the patients had visited the hospital within two days since the onset. The risk of hospitalization from pneumonia in influenza-positive patients (0.5%, N = 1/212) was identical to that in influenza-negative patients (1.1%, N = 3/264; p = 0.6).

**Table 1:** Characteristics of the study patients by case-control status.

	Influenza A rapid test positive case	Influenza B rapid test positive case	Influenza A/B rapid test negative control	P value^b^
	N = 198	N = 16	N = 264	
	N (%)/Median (IQR^a^)	N (%)/Median (IQR)	N (%)/Median (IQR)	
Sex				
Female	105 (53)	11 (68.8)	128 (48.5)	0.3
Male	93 (47)	8 (31.2)	136 (51.5)	
Age category				
15–19 years	15 (7.6)	6 (37.5)	27 (10.2)	0.1
20–49 years	132 (66.7)	10 (50.0)	161 (61)	
50–64 years	33 (16.7)	0 (0)	32 (12.1)	
> = 65 years	18 (9.1)	0 (0)	44 (16.7)	
Age (year)	34.5 (24)	21 (14)	36 (30.5)	0.1^c^
Chronic conditions				
Present	62 (31.3)	2 (12.5)	92 (34.9)	0.3
Absent	136 (68.7)	14 (87.5)	172 (65.1)	
Smoking				
Current/ex smoker	47 (23.7)	3 (18.8)	61 (23.1)	1
Non smoker	87 (43.9)	12 (75)	121 (45.8)	
Unknown	64 (32.3)	1 (6.2)	82 (31.1)	
Alcohol				
Drink	57 (28.8)	5 (31.3)	87 (33)	0.5
Not drink	77 (38.9)	10 (62.5)	97 (36.7)	
Unknown	64 (32.2)	1 (6.3)	80 (30.3)	
Date of OPD visit				
Dec 20–31 2010	23 (11.7)	1 (6.3)	21 (8)	<0.001
Jan 2011	102 (51.5)	1 (6.3)	78 (30)	
Feb 2011	58 (29.3)	2 (12.5)	67 (25.4)	
Mar 2011	14 (7.1)	4 (25)	52 (19.7)	
Apr 2011	1 (0.5)	8 (50)	46 (17.4)	
Body temperature (°C)				
37.1–37.9	59 (30)	1 (6.2)	75 (28.6)	0.9
38.0–38.9	97 (49.2)	12 (75)	137 (52.3)	
39.0–	41 (20.8)	3 (18.8)	50 (19.1)	
Duration of symptoms (days between onset and rapid test)				
0–1	133 (67.2)	7 (43.8)	189 (71.9)	<0.001
2–3	62 (31.3)	8 (50)	53 (20.2)	
4–5	3 (1.5)	1 (6.2)	21 (8)	
Received influenza vaccine for 2010/11 season				
Vaccinated	47 (23.7)	3 (18.8)	105 (39.8)	<0.001
Unvaccinated	127 (64.1)	12 (75)	119 (45.1)	
Unknown	24 (12.1)	1 (6.2)	40 (15.2)	

a Interquartile range.

b Chi-squared tests were performed comparing influenza A- and/or B-positive cases and influenza-negative controls otherwise indicated.

c T-test.

The vaccination history was recorded in 86% of enrolled patients. Among the control patients with vaccination history, 46% (N = 105/224) were immunized for influenza: 44% (N = 72/164) in aged <50 years and 55% (N = 33/60) in aged > = 50 years. The precise date of vaccination was not available in our study; instead, the month of vaccination was recorded for approximately 80% of the vaccinated participants. 14.7% (N = 17/116) of the vaccinated patients had received the vaccine within two months of the clinic visit. Because our information on the timing of vaccination was limited, it was not included in our analyses.

The estimated VEs against medically attended influenza are shown in Table 2. The patients' gender was not included in the final models because its inclusion did not change the magnitude of the effect. The TIV reduced the risk of medically attended influenza by 60.9% in the patients who were 15–49 years of age, but the reduction was not significant for the patients who were > = 50 years of age (test for interaction, p = 0.2). In our sensitivity analysis, there was only a minimal effect on the adjusted VE estimates against influenza when using the complete-case analysis (1.9% difference).

**Table 2:** Vaccine effectiveness (95% CI) of the trivalent influenza vaccine against medically attended influenza in Nagasaki, Japan, December 2010–April 2011.

	Primary analysis	Complete-case analysis
	(95% CI)	(95% CI)
Unadjusted	58.6 (37.2 to 72.7)	58.6 (37.2 to 72.7)
Adjusted	47.6 (16.4 to 67.1)^a^	45.7 (5.6 to 68.7)^a^
Restricted to those aged 15–49 years	60.9 (31.3 to 77.8)^a^	56.4 (19.6 to 76.4)^a^
Restricted to those aged 50 years and over	−52.6 (−306.5 to 42.7)^a^	−37.3 (−658.7 to 75.2)^a^
Restricted to those with no chronic condition	50 (9.4 to 72.4)^b^	50.5 (4.1 to 74.4)^b^

a Adjusted for age group, chronic conditions, month of visit, duration of symptom, smoking and alcohol.

b Adjusted for age group, month of visit, duration of symptom, smoking and alcohol.

## Discussion

According to this influenza VE study using RIDT, the estimated VE of TIV against medically attended influenza among adult Japanese population was 47.6% during the 2010/11 season in our setting. The TIV reduced the risk of medically attended influenza by 60.9% for patients less than 50 years of age, but a significant reduction was not observed for patients who were 50 years of age and older. Although our study is limited by the use of RIDT to classify the influenza cases without performing confirmatory testing, our estimates are comparable with those from recent reports in different settings.

The effectiveness of the MIV against medically attended influenza A (H1N1) 2009 during the 2009/10 season was 72% in Europe [24] and 93% in Canada [6]. Recent studies have demonstrated that the VE of TIV against influenza A (H1N1) 2009 was 79% in Australia (2010 season) [25] and 58% (2010/11 season) in Spain [26]. Our overall VE was lower than those estimates. Several factors explain this. First, the RIDT that was used in our study was not as specific as RT-PCR. The sensitivity and specificity of the RIDT against influenza A (H1N1) 2009 were 60–70% and 84–99%, respectively, in community settings [19], [27]. Simulation studies suggest that the use of an imperfect test to diagnose influenza underestimates the true VEs [16], [17]. Thus, our VE estimates must be considered as minimum values. Second, according to the national surveillance report, 53% of the laboratory-confirmed influenza A was A (H1N1) 2009 and 32% was H3N2 in the 2010/11 season [28]. An Australian study demonstrated that the VE of TIV against H3N2 may have been lower than that of the MIV against A (H1N1) 2009 [5]. Therefore the subtype distribution should be considered in interpreting our estimate. Third, our VE estimate among the patients who were > = 50 years of age was substantially lower than the estimate among the younger adults. Although the statistical power was low due to the limited number of elderly, the finding was compatible with those results from previous studies of the MIV against influenza A (H1N1) 2009 in Canada (Manitoba) [29], USA [30] and the Netherlands [31]. The benefit of the seasonal influenza vaccine for elderly people has not been fully established [1], [32], [33]. The age distribution of study participants may have affected the overall VEs.

Above comparisons with previous reports indicate that our influenza VE estimates are reasonably accurate and sufficient for a clinical/local use. Despite its low sensitivity and specificity, RIDT has overwhelming advantages in the VE study [17]. Testing many samples by RT-PCR is costly and is not accessible for the majority of clinicians, whereas RIDT is widely used in daily practice and is covered by insurance in Japan because the RIDT results often influence the clinicians to prescribe antiviral drugs such as oseltamivir. Participants are not required to receive additional invasive testing or charges for the studies. Therefore, if the test-negative design is combined with RIDT, a sufficient sample size can be rapidly obtained without any additional cost, even in a single-institutional design like our case. We believe that such dispensary-based information on influenza VE is very useful for clinicians to promote vaccination for their patients and communicate with other health experts. It may be also useful for infection control in hospital personnel.

On the other hand, this study has limitations due to a nature of the observational study design which is prone to bias and confounding [34], [35]. Our patients were enrolled by clinicians who were aware of their vaccination status, and it caused a selection bias. The characteristics of the patients with ILI who were not tested by RIDT were distinct from those of our patients tested by RIDT. However, more than 90 percent (N = 526/570) of the ILI patients who visited our hospital were tested by RIDT, and the vaccination coverage among our control patients was identical to the national estimate (45%). We thus believe that this bias did not largely change our VE estimates. History of vaccination was taken only through the questionnaire and electronical medical records and was not validated. Biases in reporting, if any, may have also affected the VE estimate. The hospital vaccination records are, if available, preferably used to validate the vaccination history to minimize recall bias. In our multivariable analysis, unmeasured confounding factors may have remained. In the younger age group, the better educated people may have got vaccinated and the better education may have resulted in less exposure to the disease; therefore, it may have appeared as if they were protected by immunization. Also socially active older adults may have taken the vaccines because they knew that they were at an increased risk, and it may have appeared as if the vaccine had negative effect. Socioeconomic status must be considered in future studies.

In addition, clinicians who plan to introduce this method in their clinics or hospitals must pay attention in interpreting the VE estimates. The VE estimated from this design is a rapid estimate which may be usable in a local setting and should not be extrapolated to provincial or national level. The RIDT-based VE estimates tend to underestimate the true VEs due to the low accuracy of rapid tests [16], [17], and the sensitivity and specificity of RIDT may differ across seasons. The age-stratified VE estimates may be limited by small sample size in a single-institutional study design. Another limitation is that this method gives only VE estimates against medically attended influenza but not against influenza disease. The true influenza VEs must be confirmed by expert agencies with more rigid study design using accurate testing such as RT-PCR and viral culture.

### Conclusions

The test-negative case-control design with RIDT is feasible in clinical settings and provides VE estimates against medically attended influenza. Although it is not a perfect design, this simplified method can provide timely influenza VE estimates which must be useful for clinicians and patients.
